# An Intelligent Customer-Driven Digital Solution to Improve Perioperative Health Outcomes Among Children Undergoing Circumcision and Their Parents: Development and Evaluation

**DOI:** 10.2196/52337

**Published:** 2024-02-16

**Authors:** Zhi Yin Kwa, Jinqiu Li, Dale Lincoln Loh, Yang Yang Lee, Guangyu Liu, Lixia Zhu, Minna Pikkarainen, Honggu He, Vidyadhar Padmakar Mali

**Affiliations:** 1 Department of Nursing National University Hospital Singapore Singapore; 2 National University Health System Singapore Singapore; 3 Nursing Department, Zhuhai Campus Zunyi Medical University Zhuhai China; 4 Alice Lee Centre for Nursing Studies Yong Loo Lin School of Medicine National University of Singapore Singapore Singapore; 5 Department of Paediatric Surgery National University Hospital Singapore Singapore; 6 Integrated Health Promotion Ministry of Health Office for Healthcare Transformation Singapore Singapore; 7 Hôpital Chinois de Montréal Centre Intégré Universitaire de Santé et de Services Sociaux du Centre-Sud-de-l'Île-de-Montréal Montreal, QC Canada; 8 Department of Health Technology and Rehabilitation and Department of Product Design Oslo Metropolitan University Oslo Norway; 9 Martti Ahtisaari Institute Oulu Business School University of Oulu Oulu Finland

**Keywords:** circumcision, self-efficacy, perioperative anxiety, postoperative pain, mobile phone, postoperative, pain, anxiety, distractions, distraction, perioperative, interview, interviews, child, children, surgery, surgical, recovery, health outcome, health outcomes, pediatric, pediatrics, content analysis, emotional, mobile health, app, apps

## Abstract

**Background:**

Circumcision as a common elective pediatric surgery worldwide is a stressful and anxiety-inducing experience for parents and children. Although current perioperative interventions proved effective, such as reducing preoperative anxiety, there are limited holistic solutions using mobile apps.

**Objective:**

This paper aims to describe the development and primary evaluation of an intelligent customer-driven smartphone-based app program (ICory-Circumcision) to enhance health outcomes among children undergoing circumcision and their family caregivers.

**Methods:**

Based on the review of the literature and previous studies, Bandura’s self-efficacy theory was adopted as the conceptual framework. A multidisciplinary team was built to identify the content and develop the apps. Semistructured interviews were conducted to evaluate the ICory-Circumcision.

**Results:**

The ICory-Circumcision study was carried out from March 2019 to January 2020 and comprised 2 mobile apps, BuddyCare app and Triumf Health mobile game app. The former provides a day-by-day perioperative guide for parents whose children are undergoing circumcision, while the latter provides emotional support and distraction to children. In total, 6 participants were recruited to use the apps and interviewed to evaluate the program. In total, 4 main categories and 10 subcategories were generated from content analysis.

**Conclusions:**

ICory-Circumcision seemed to lean toward being useful. Revisions to ICory-Circumcision are necessary to enhance its contents and features before advancing to the randomized controlled trial.

**Trial Registration:**

ClinicalTrials.gov NCT04174404; https://clinicaltrials.gov/ct2/show/NCT04174404

## Introduction

### Background

Male circumcision is a surgery to remove the foreskin of the penis [1]. It is one of the most common day pediatric surgeries worldwide, with an estimated 30% incidence of circumcised males, of which two-thirds were Muslim [2,3]. Singapore’s male circumcision prevalence is about 15% [4]. Male circumcision is commonly performed in Singapore between ages 8 and 11 years often for religious reasons [3]. With Muslims comprising 14.7% of Singapore’s population [5], circumcision is likely common in Singapore.

While global and local trends can be ascribed to mainly religious or cultural reasons [6], it is also expected to rise due to growing evidence of health benefits such as up to 73% protection against acquiring HIV [7,8] and reduced risks of urinary tract infections [4]. Voluntary medical male circumcision may save US $16.5 billion by 2025 from averted HIV treatment and associated costs [8].

Rising preferences for elective male circumcision may also be explained by low complication rates of 0% to 30% in male circumcision [9] and benefits of elective day surgeries such as reduced hospital-acquired infection risks, financial burdens, and disruptions to daily commitments like school [10]. With shifts in male circumcision being done as elective surgeries, parents have to assume heavier parenting roles as their involvement in perioperative care increases [11]. These tasks include managing their child’s preoperative fasting and postoperative wound. Despite the advantages of elective male circumcision, surgeries, even minor ones, are still stressful and anxiety-inducing periods for both parents and children [12,13]. The unfamiliarity of settings and perioperative care and fear of their child’s death are reasons for parental preoperative anxiety [11,14]. In fact, parents of children who undergo day surgery have been found to experience higher parental preoperative anxiety than parents of hospitalized children [15]. This could be due to increased responsibilities and inadequate time to adjust to unfamiliar settings [11]. High parental preoperative anxiety often results in unfavorable somatic symptoms such as insomnia that can hinder parents’ everyday functions and impact work productivity [16]. Parental preoperative anxiety and lack of knowledge can incur unnecessary costs for families and hospitals through unnecessary visits to the emergency department after male circumcision [13,17]. Furthermore, parental preoperative anxiety affects children’s emotional responses and increases children’s preoperative anxiety, as children heavily depend on their parents, especially during foreign events like surgery [11,18,19]. Up to 84% of children undergoing male circumcision had experienced fear or worry, suggesting children’s preoperative anxiety is prevalent in pediatric male circumcision [20]. Children’s preoperative anxiety has been correlated with consequences such as increased postoperative pain, sleep-related problems, and hindered recovery [21-23]. Children’s preoperative anxiety also causes prolonged induction and further use of sedatives and requires additional nursing staff, incurring more costs for families and hospitals [13,24-26].

These combined findings suggested the need for a more comprehensive and effective solution to decrease children’s preoperative anxiety. This study aimed to develop an intelligent customer-driven solution for pediatric surgery care on the improvement of outcomes of parents and their primary school-aged children undergoing circumcision (ICory-Circumcision) and examine the feasibility of the program.

### Review of Current Circumcision Clinical Practice in Singapore

**Figure 1:**
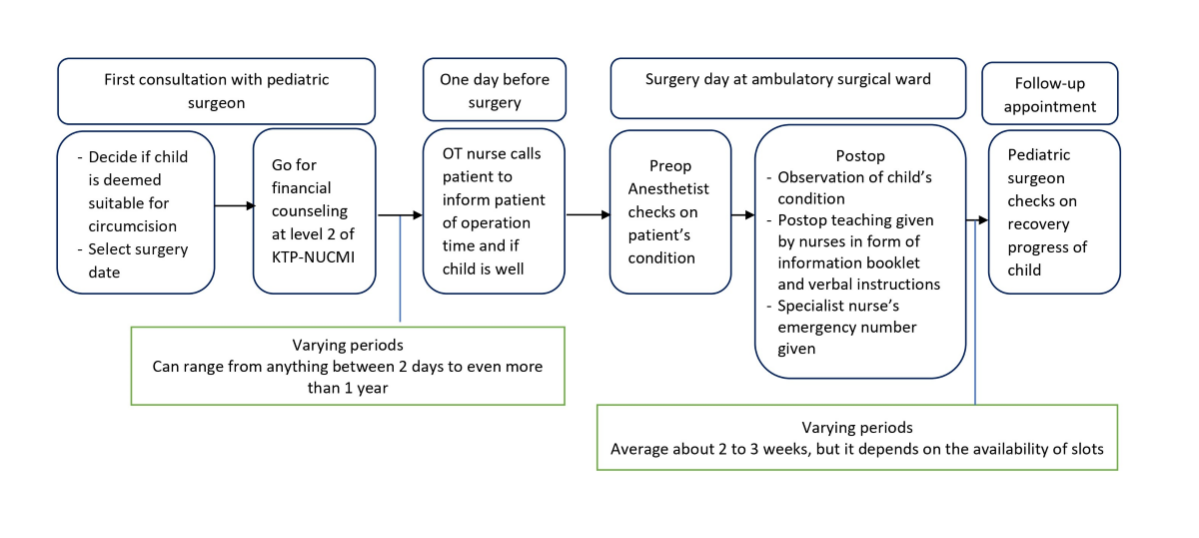
Pediatric circumcision routine care. KTP-NUCMI: Khoo Teck Puat—National University Children’s Medical Institute; OT: operation theater.

Figure 1 shows the current pediatric circumcision routine care in the Singapore health system. At one of Singapore’s tertiary hospitals, about 12% of children who underwent male circumcision reverted to the emergency department before scheduled follow-up appointments [27]. However, only 2% of these children had postoperative problems that warranted medical intervention, while the remaining 10% did not require specialist care and, therefore, were avoidable [27]. As seen, parents’ lack of postoperative knowledge and communication with health care professionals (HCPs) led to what could have been avoidable costs. Additionally, parents in Singapore have expressed the desire for information provision through mobile apps [14]. Current practices of providing surgery-related information for male circumcision are through verbal or written mediums. Technological-based solutions have yet to be incorporated. Therefore, incorporating ICory-Circumcision into pediatric male circumcision settings in Singapore could potentially save resources for families and hospitals.

### Review of Literature and Findings From Previous Studies

Parents and children undergoing elective surgeries experience stress and negative emotions [28]. As a result, studies have been conducted to explore their needs. Parents desire emotional support and perioperative information, involvement in their child’s perioperative care, and building good collaboration with HCPs [29-33]. Studies have shown that providing information helped decrease parental preoperative anxiety and encouraged parents’ participation in their children’s care [28,32,34]. Parents wished to know surgery indications, medications and fasting instructions, involvement in the operating theater, and pain and wound management [35-37]. Their strong desire for such information could be attributable to their major roles in assimilating information to their children [38]. Parents also hoped for such information to be individualized and disseminated to them via web-based mobile apps or literature [14,29,36]. They also preferred if postoperative information was given before surgery instead of just before discharge [39].

Children desire preparational information and tend to seek help from parents when they experience pain [40,41]. The majority of the children experience moderate to severe pain during the postoperative period despite their parents’ involvement in care [40]. This could suggest that pain was undertreated [33]. This further highlights the need to provide education on pain management to parents. Children desired parental presence and more distraction techniques to be used by their parents for pain management [41]. Parents also wished to monitor their children’s pain in addition to the strategies [31]. Finally, children hoped for more communication between parents and nurses to assist with postoperative pain [42].

Various technological-based interventions have been developed and aimed at parents of children undergoing elective surgeries. Videos aimed at educating parents about their children’s surgery have been used in several studies. However, the contents of the studies varied rather widely. Chow et al [43] conducted a systematic review and found that videos that included both preoperative and postoperative information were more effective. Two such studies focused on perioperative education; however, only one study showed a decrease in parental preoperative anxiety, while the other showed no significant changes [17,44]. The video contents of 2 studies were about the surgery day [45,46]. Chartrand et al [45] aimed to educate parents about the experience in the recovery room, and it improved parents’ knowledge but not anxiety. Berghmans et al [46] aimed at modeling a hospital tour for parents and children, but no significant changes in parental anxiety. Other studies focused on different surgery periods such as informed consent and postoperative pain management [47,48]. Two studies examined the effects of web-based preparation programs for parents and children undergoing elective surgeries, and both were effective in reducing parental preoperative anxiety [49,50]. Both interventions had elements of surgery-related educational modules for both parents and children, and both studies were effective in reducing parental anxiety. Children’s preoperative anxiety decreased in Fortier and Kain’s study [49] but did not in Wright et al’s study [50]. SMS text messages and mobile apps were also used in several recent studies in pediatric surgery settings [51-55]. Four studies used SMS text messages to convey perioperative education to parents of children undergoing elective surgeries [51-53,56]. These studies allowed real-time communication with HCPs via SMS text message or phone call. The programs were able to decrease parental preoperative anxiety, increase parental knowledge, reduce children’s preoperative anxiety, and improve parent satisfaction, which resulted in neither operation cancellations nor visits to the emergency department. While those 4 studies had no intraoperative texts, Kwan et al [57] examined the effectiveness of sending intraoperative texts, and it was effective in reducing parental anxiety. Ji et al [54], on the other hand, developed an app that uses drawings to explain procedures to parents, which resulted in reduced parental preoperative anxiety and improvement in parental satisfaction. Bailey et al [55] tested the effects of an educational video app on perioperative information and parents’ role in the operating theater.

Several studies have examined the effectiveness of mobile game apps on children’s preoperative anxiety [58-61]. These 4 studies used game apps that were available in app stores and were selected based on age appropriateness. There was a significant reduction in children’s preoperative anxiety after the children played the games in 3 studies. In addition, Cumino et al [58] also showed that a combination of strategies (parental leaflet+mobile game) was more effective in lowering the prevalence of anxiety in the operating room. Marechal et al [61] showed no significant difference in children and parental anxiety. A few studies also used mobile apps to prepare children for surgery [62-64]. All 3 studies aimed to simulate the operating room but through different presentations in the apps: medical clowning video, multimedia app presenting hospital procedures in stages and accompanying videos, and photographs and cartoons. All 3 studies led to a significant decrease in children’s preoperative anxiety. Fernandes et al [63] also showed decreased parental state anxiety.

Our review of the literature showed that perioperative needs of parents and children undergoing elective surgeries have been extensively researched, and as a result, many interventions have been developed to address their needs. However, there is a lack of technological-based interventions targeted at parent’s self-efficacy in children’s perioperative care. There is also a dearth of studies using mobile app–based education for parents, and none were conducted in Singapore.

## Methods

### Content Development and Theoretical Framework

**Figure 2:**
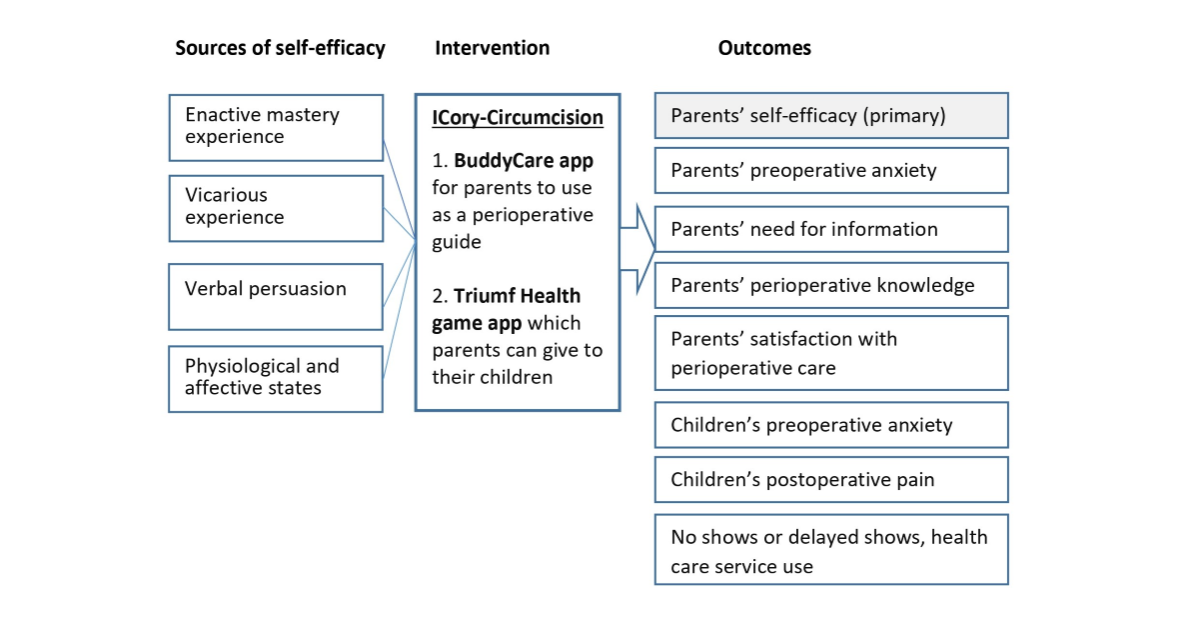
Theoretical framework used for the study.

Taking all the gathered information into consideration, Bandura’s self-efficacy theory and interrelationships between self-efficacy, anxiety, knowledge, and satisfaction were adopted as the theoretical and conceptual framework to guide the development of ICory-Circumcision and methodology of this study (Figure 2).

Bandura posited that self-efficacy is derived from the integration of information from 4 sources of self-efficacy, namely, enactive mastery experience, vicarious experience, verbal persuasion, and emotional and physiological states [65]. Enactive mastery experience refers to parents’ prior experiences in taking care of children throughout the perioperative period, and it is the most influential source of self-efficacy. Vicarious experience was gained by observation and modeling, as it offers parents chances to judge their abilities against a reference point to master tasks [65]. Verbal persuasion refers to persuasive information parents receive from others to enhance parental self-efficacy. Emotional and physiological states influence self-efficacy as a person’s functions are affected [65].

Parental self-efficacy has been shown to negatively correlate with anxiety and child distress and positively correlate to child cooperation [66,67]. High parental preoperative anxiety has been positively correlated with children’s preoperative anxiety, while children’s preoperative anxiety has been positively correlated with higher postoperative pain [68-70]. Additionally, parental preoperative anxiety has been reported to increase the likelihood of surgical cancellations due to lower compliance with fasting instructions [70]. Based on Bandura’s theory, anxious parents could lower parental self-efficacy and subsequently affect children’s perioperative outcomes such as children’s preoperative anxiety and postoperative pain [71]. Parental preoperative anxiety has been shown to be positively correlated to the need for information, thus further reinforcing the need to develop interventions to provide the information parents require [72].

### ICory-Circumcision Components in Relation to Self-Efficacy Theory

**Figure 3:**
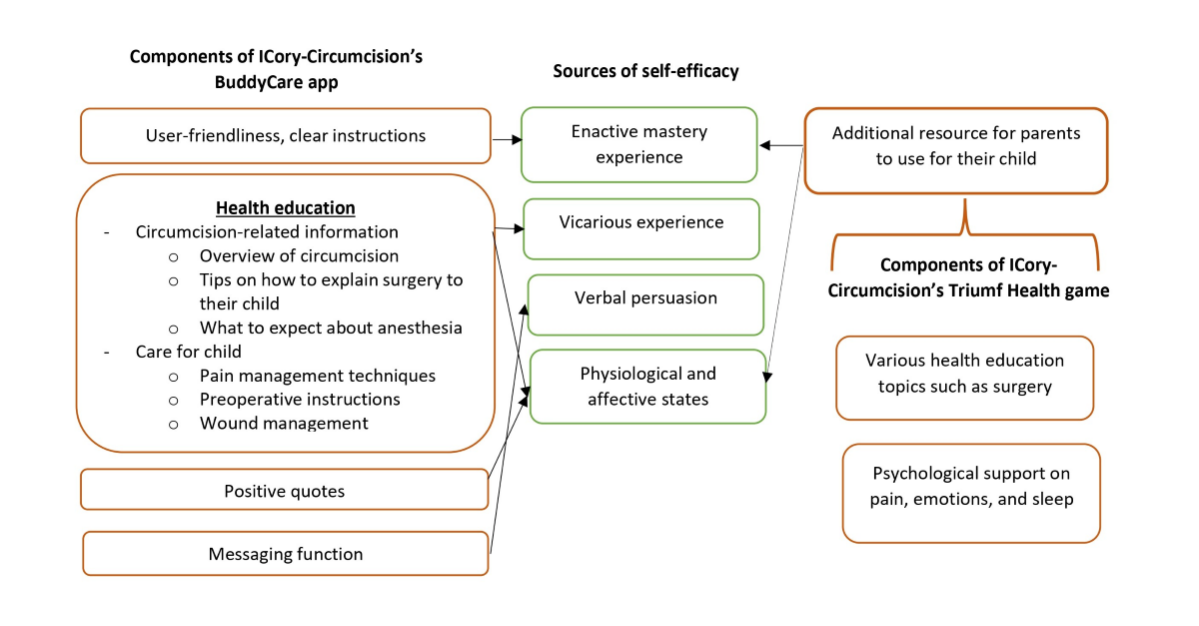
ICory-Circumcision components in relation to self-efficacy theory.

The Template for Intervention Description and Replication (TIDierR) checklist and guide was also recommended to be used in the process of intervention development [73]. The components of ICory-Circumcision in relation to the self-efficacy theory are depicted in Figure 3.

### Qualitative Evaluation of the ICory-Circumcision Program

A self-developed interview guide for field test of BuddyCare and TriumfHealth apps (Multimedia Appendix 1) was used to guide the semistructured interviews to explore the perceptions of the strengths, weaknesses, and the use of ICory-Circumcision from parents, children, and HCPs who used ICory-Circumcision. The qualitative data obtained from process evaluation were analyzed using inductive content analysis [74,75]. The analysis was done in 3 phases: preparation, organizing, and reporting [74], while steps were taken to achieve trustworthiness [76].

### Ethical Considerations

Ethics approval (2019/00582) and amendment approval were obtained from the National Health Group Domain Specific Review Board before the commencement of the study. All research team investigators obtained the Collaborative Institutional Training Initiative certificate. Informed consent was obtained from the children’s parents, while assent was taken from the children. All potential participants were given information about the study using the participant information sheet to inform them about the study’s aim, potential benefits, risks, and responsibilities. Voluntary participation, the right to withdraw, and confidentiality were highlighted. Informed consent was not obtained from the HCPs who were interviewed as they were part of the study team. All data from questionnaires were entered electronically into the study hospital’s REDCap (Research Electronic Data Capture; National University Hospital) database, and the data were exported as nonidentifiable data into SPSS (IBM Corp) for data analysis. Only identified study team members with intranet access were able to enter, monitor, and export data. The audio recordings of the interview will be deleted from the audio recorder and stored in the principal investigator’s password-protected computer in the office of Alice Lee Centre for Nursing Studies. All physical records such as consent forms and questionnaires were stored in a locked cupboard at the Department of Pediatric Surgery in National University Hospital. The documents and electronic data will be destroyed after 6 years upon closure of the study by the Domain Specific Review Board. A brand-new SIM card was purchased for the study phone, and it will be disabled and destroyed at the end of the study as well. In addition, no identifiable information was entered in ICory-Circumcision’s apps; instead, pseudonyms and precreated emails were used. This ensured that no participant identifiers were captured by the apps’ companies to protect the participants’ privacy and data confidentiality. No compensation in terms of material or financial benefits was provided to the research participants who participated in this program.

## Results

This study was carried out from March 2019 to January 2020 and comprised 2 mobile apps.

### BuddyCare Mobile App for Parents

**Figure 4:**
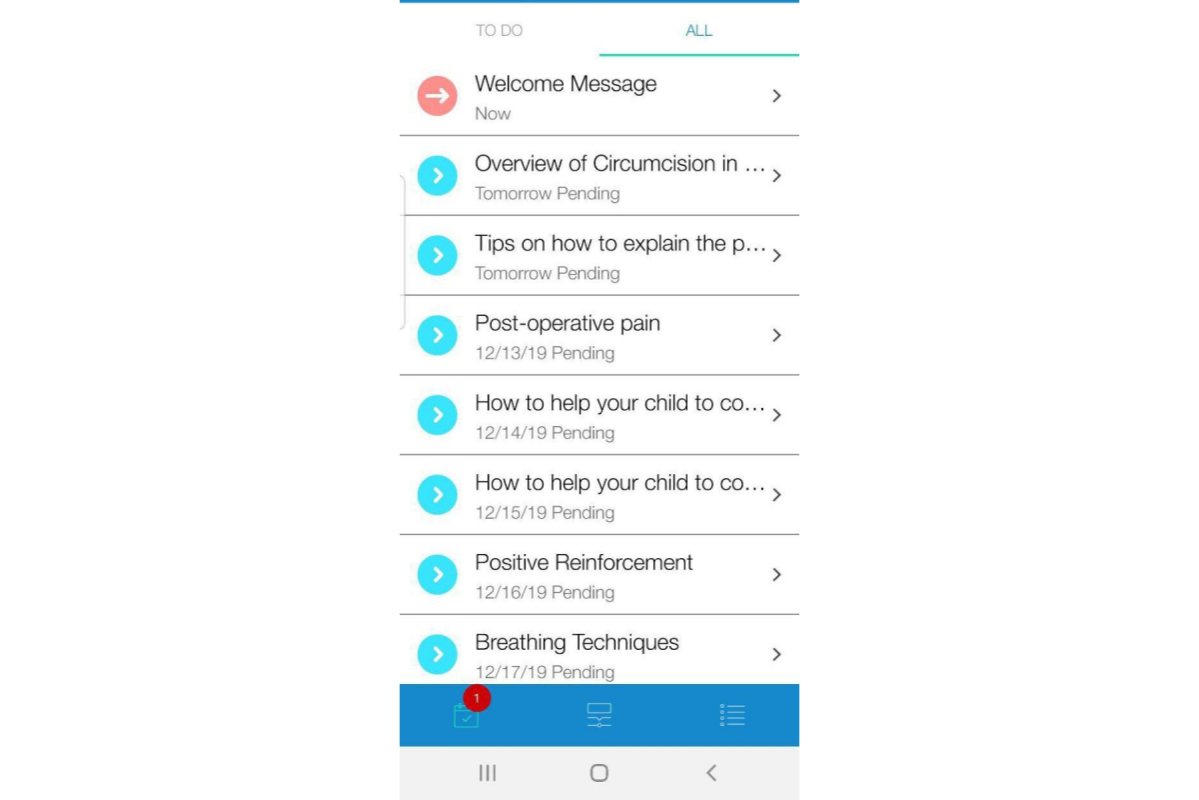
Screenshot of BuddyCare overview.

**Figure 5:**
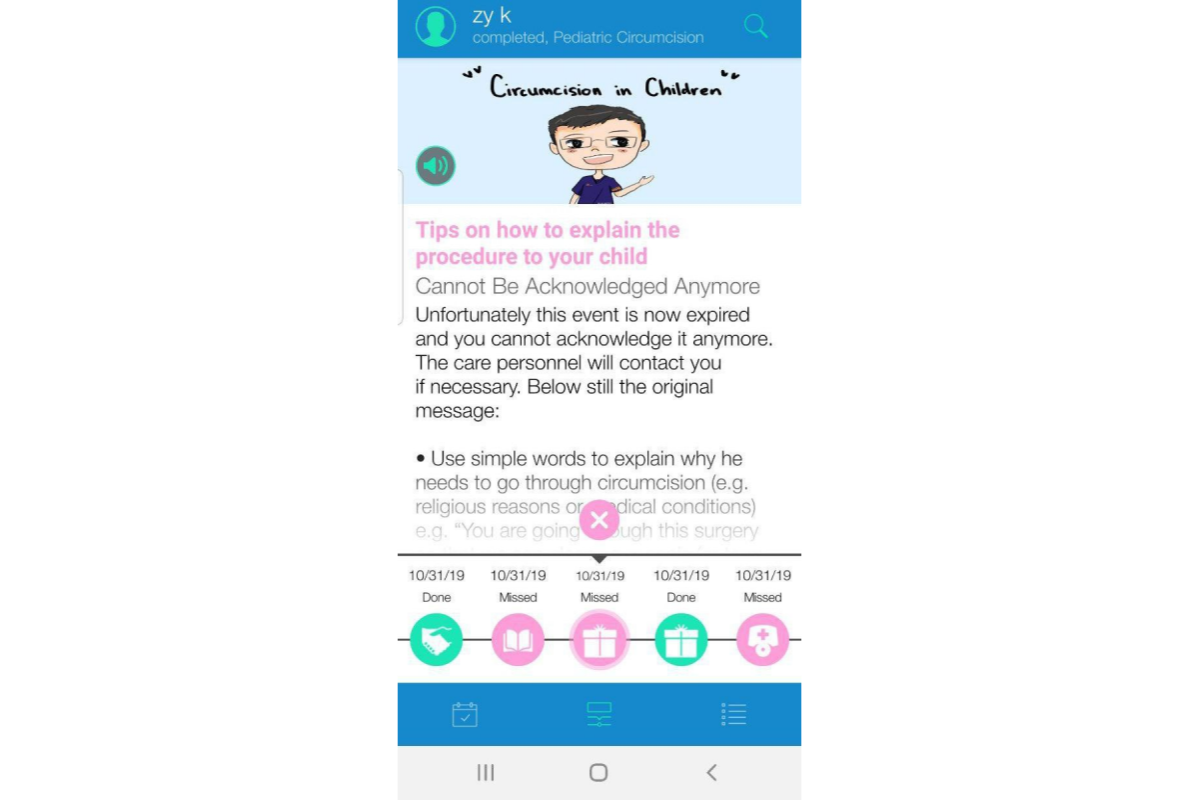
Screenshot of BuddyCare care for parents.

One of the eventual products was the BuddyCare mobile app that provides a comprehensive day-by-day perioperative guide for parents regarding their children’s surgery with an interface to communicate with HCPs. Parents were able to select the surgery date and time on the app, and then, the contents were arranged according to each participant’s timeline. The timeline of BuddyCare contents can be found in Multimedia Appendix 2. Two educational topics on the app were selected in accordance with the parental and children’s needs in the literature review and surgery pathway, one is circumcision-related information, including an overview of circumcision, tips on how to explain the surgery to their children, and what to expect about anesthesia; another one is caring for children, including pain management techniques (eg, emotional support, breathing techniques, positive reinforcement, and distraction), preoperative instructions (eg, fasting instructions), and wound management (eg, how to clean and when to bring their child to the emergency department; Figures 4 and 5). Positive quotes are refreshed periodically as emotional support to motivate the parents throughout the perioperative process (Multimedia Appendix 3). With the messaging function, participants are able to communicate with HCPs by sending SMS text messages through the messaging tab (Multimedia Appendix 4). The HCPs in the study team will be able to access the SMS text messages via a BuddyCare dashboard, and they can reply to the participant through this dashboard.

### Triumf Health Mobile Game App for Children

**Figure 6:**
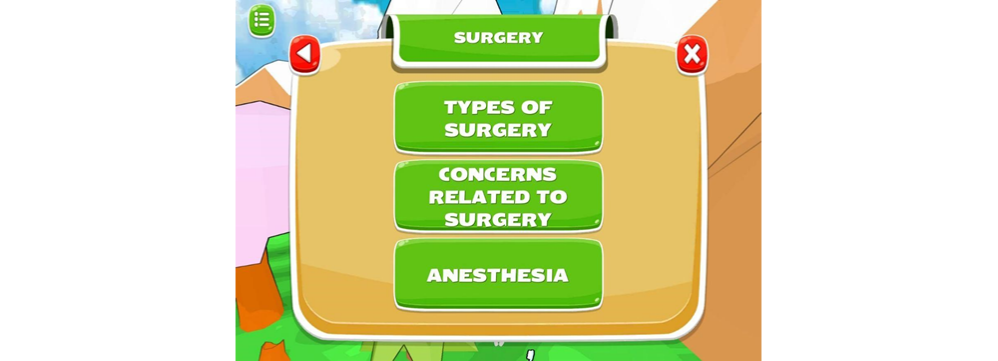
Screenshot of Triumf Health contents.

Another product was the Triumf Health mobile game app that provides emotional support and distraction to children. The game allowed the children to customize their own characters and save Triumfland city from a diseased monster by finding one’s inner superpowers. The child was able to control their character to venture around Triumfland and gain points through completing quests in order to help the town doctor to eradicate the disease monster. One important aspect of the game was providing general surgery information to the child (Figure 6). The child could access the topics at any time on their own volition, and the information about each topic was displayed in levels to cater to the child’s reading and comprehension ability. To illustrate, once the child accessed the information in level 1, the information would be presented, and the app would prompt the child to ask if he understood the information. If the child says no, a short summary of the information from level 1 will be presented in short simple sentences. The app also rendered various psychological support to the children such as pain and mood. If the child responded with unfavorable answers such as severe pain or a negative emotion, the game provided appropriate words of encouragement to the child (Multimedia Appendix 5). The abovementioned features of the app made Triumf Health game user experience personalized and dynamic. Further gameplay, that is, accessing the educational module, entertainment games, and other elements of the intervention, was determined by the in-game choices made by the player. Furthermore, the provision of psychological support is dynamically dependent on the patient’s individual progress and in-game progress.

### The Qualitative Evaluation on ICory-Circumcision

In total, 6 participants (2 boys who were going to take male circumcision, 2 of their parents, and 2 HCPs) were recruited to use the apps and were required to share their perceptions about the apps. An interview guide was developed and followed (Multimedia Appendix 1). In total, 4 main categories and 10 subcategories were generated from content analysis and presented in Textbox 1.

**Categories and subcategories.**

**Strengths of ICory-Circumcision**

- BuddyCare content is useful
  Comprehensive (n=3) and easy to understand (n=1)
  Learning experience for parents (n=3)
  Useful especially for parents with no experience (n=2)
- Mobile apps as useful platform
  Convenient (n=2) and appropriate for the modern era (n=2)
  BuddyCare supports routine care (n=4)
- Reasons for liking the Triumf Health game app
  Follow-up on child’s postoperative status (n=1)
  Enjoyed the game and its features (n=1)

**Factors for dissatisfaction in ICory-Circumcision**

- Reasons for disliking the Triumf Health game app
  Boring (n=3) and frustrating (n=1)
  Children preferred other means of distraction (n=4)
- Communication issues
  Delayed and unsatisfied response in Buddycare (n=1)
  Inconvenience of BuddyCare dashboard (n=1) and difficulty in using (n=1)

**Outcomes of using ICory-Circumcision**

- Opinions of BuddyCare on perioperative outcomes
  Reduction in parental and child anxiety (n=2)
  Improved parental confidence in taking care of the child (n=1)
- Opinions of Triumf Health on perioperative outcomes
  Minimal help in managing preoperative anxiety (n=3)
  No help with coping with postoperative pain (n=1) versus little help (n=1)

**Suggestions for improvement**

- BuddyCare content suggestions
  Less words (n=1)
  Different languages for important information (n=1)
  More visuals (n=2) versus sufficient visuals (n=1)
- BuddyCare technical aspects
  Reduce reminders (n=1) versus adequate reminders (n=1)
  Making a dashboard app (n=1)
- Fidelity of ICory-Circumcision
  Training for health care professionals (n=2)
  Intervention delivery suggestions (n=2)

## Discussion

### Main Findings

The principal aim of the program was to develop an intelligence solution to increase parental self-efficacy and decrease parental and children’s preoperative anxiety. Parents generally expressed positive reactions toward the BuddyCare app. They found BuddyCare to be comprehensive, convenient, and useful, and they would highly recommend it to other parents. Triumf Health app was useful in follow-up postoperative pain and emotional care for children. These findings align with the aims of ICory-Circumcision and the HCPs’ views. Participants also found ICory-Circumcision to be a good resource that complements routine care, which is similar to another study [49].

Feedback on Triumf Health and BuddyCare should be taken and revise ICory-Circumcision as an intervention. Based on the mixed reactions from the qualitative interview, it may suggest that ICory-Circumcision may not be individualized enough for participants. For example, more visuals such as videos could be added into BuddyCare, but they could be placed in a different tab, which allows parents the liberty to access that section or not. This is to cater to the different levels of comfort each parent has with seeing pictures of open wounds. For Triumf Health, the number of words could be reduced, and the mechanics of the game could be reviewed with the team in Finland to see if it could be better improved to suit the needs of the children in Singapore.

Lack knowledge of pain management strategies and wound management techniques could affect the development of parental self-efficacy and increase negative emotions [29,72]. Studies showed that providing information about their children’s surgery to parents could reduce parental preoperative anxiety and showed an increase in parental self-efficacy [28,55,77]. Past experiences could have contributed to the high parental self-efficacy. Bandura [65] suggested that mastery experiences have the strongest influence on self-efficacy out of the 4 sources, and if caregivers had previous caregiving experience, they had high parental self-efficacy. On the contrary, parents would have higher anxiety when they have the first surgical experience due to medical reasons, which could impede the self-efficacy gained from physiological and affective states [65]. Therefore, providing adequate knowledge to parents is an efficient way to improve health-related outcomes.

Mobile apps are ubiquitous among parents and children, possibly due to the convenience brought by their easy accessibility [78-80]. The infiltration of mobile apps into pediatric settings is clear with the advent of mobile apps aimed at helping children with different health conditions [81,82]. Therefore, the number of mobile resources HCPs have access to has greatly expanded, improving efficiency and productivity [83]. Evidently, mobile apps have tremendous potential as a platform for information delivery.

As there are limited interventional studies presenting the development process, this study will contribute to the body of literature about intervention development [84]. This informs readers about the possible challenges that one can encounter should they decide to embark on similar intervention development [73]. This study also provided insights into the feasibility of ICory-Circumcision and the study’s methodology, which could improve the main trial’s processes and prove the effects of ICory-Circumcision. If the effects are then proved, it could potentially save nurses’ time, as nurses are heavily involved in providing education to parents and children about surgery [17,34]. Although our qualitative evaluation of ICory-Circumcision involved various users, including children, parents, and HCPs, the sample size was small due to the limited time for an honors student’s project and the COVID-19 pandemic occurrence in November 2019.

### Conclusions

This paper detailed the development of a holistic technology–based intervention for parents and their children undergoing elective circumcision and examined its preliminary feasibility and evaluation. The qualitative evaluation identified strengths, weaknesses, and suggestions for improvement concerning ICory-Circumcision, suggesting its potential usefulness for parents and children in perioperative outcomes. Prior to proceeding with the randomized controlled trial, revisions to ICory-Circumcision to enhance its contents and features are recommended.

## Appendix

Multimedia Appendix 1 Interview guide.

Multimedia Appendix 2 Timeline of BuddyCare contents.

Multimedia Appendix 3 Screenshot of BuddyCare positive quotes.

Multimedia Appendix 4 Screenshot of BuddyCare message function.

Multimedia Appendix 5 Screenshot of Triumf Health mood evaluation.

## Abbreviations

HCP: health care professional
REDCap: Research Electronic Data Capture
TIDierR: Template for Intervention Description and Replication

## References

[1] American Academy of Pediatrics Task Force on Circumcision (2012). Male circumcision. Pediatrics.

[2] (2011). Most frequent procedures performed in U.S. hospitals. Agency for Healthcare Research and Quality.

[3] (2010). Neonatal and child male circumcision: a global review. World Health Organisation & Joint United Nations Programme on HIV/AIDS.

[4] Morris BJ, Wiswell TE (2013). Circumcision and lifetime risk of urinary tract infection: a systematic review and meta-analysis. J Urol.

[5] (2010). Singapore census of population 2010, statistical release 1: demographic characteristics, education, language and religion. Department of Statistics Singapore.

[6] Morris BJ, Wamai RG, Henebeng EB, Tobian AA, Klausner JD, Banerjee J, Hankins CA (2016). Estimation of country-specific and global prevalence of male circumcision. Popul Health Metr.

[7] Tobian AAR, Gray RH (2011). The medical benefits of male circumcision. JAMA.

[8] (2012). Voluntary medical male circumcision for HIV prevention. World Health Organisation.

[9] Hung YC, Chang DC, Westfal ML, Marks IH, Masiakos PT, Kelleher CM (2019). A longitudinal population analysis of cumulative risks of circumcision. J Surg Res.

[10] Bowen L, Thomas M (2016). Paediatric day case surgery. Anaesth Intensive Care Med.

[11] Li HCW, Lopez V, Lee TLI (2007). Psychoeducational preparation of children for surgery: the importance of parental involvement. Patient Educ Couns.

[12] Hug M, Tönz M, Kaiser G (2005). Parental stress in paediatric day-case surgery. Pediatr Surg Int.

[13] Amália de Moura L, Dias IMG, Pereira LV (2016). Prevalence and factors associated with preoperative anxiety in children aged 5-12 years. Rev Lat Am Enfermagem.

[14] Hui WJ, Pikkarainen M, Nah SA, Nah SNJ, Pölkki T, Wang W, He HG (2020). Parental experiences while waiting for children undergoing surgery in Singapore. J Pediatr Nurs.

[15] Mishel MH (1983). Parents' perception of uncertainty concerning their hospitalized child. Nurs Res.

[16] Thompson N, Irwin MG, Gunawardene WM, Chan L (1996). Pre-operative parental anxiety. Anaesthesia.

[17] Chang SF, Hung CH, Hsu YY, Liu Y, Wang TN (2017). The effectiveness of health education on maternal anxiety, circumcision knowledge, and nursing hours: a quasi-experimental study. J Nurs Res.

[18] Kain ZN, Wang SM, Mayes LC, Caramico LA, Hofstadter MB (1999). Distress during the induction of anesthesia and postoperative behavioral outcomes. Anesth Analg.

[19] Cui X, Zhu B, Zhao J, Huang Y, Luo A, Wei J (2016). Parental state anxiety correlates with preoperative anxiety in Chinese preschool children. J Paediatr Child Health.

[20] Corduk N, Unlu G, Sarioglu-Buke A, Buber A, Savran B, Zencir M (2013). Knowledge, attitude and behaviour of boys and parents about circumcision. Acta Paediatr.

[21] Kain ZN, Mayes LC, Caldwell-Andrews AA, Karas DE, McClain BC (2006). Preoperative anxiety, postoperative pain, and behavioral recovery in young children undergoing surgery. Pediatrics.

[22] Caporino NE, Read KL, Shiffrin N, Settipani C, Kendall PC, Compton SN, Sherrill J, Piacentini J, Walkup J, Ginsburg G, Keeton C, Birmaher B, Sakolsky D, Gosch E, Albano AM (2017). Sleep-related problems and the effects of anxiety treatment in children and adolescents. J Clin Child Adolesc Psychol.

[23] Das S, Kumar A (2017). Preoperative anxiety in pediatric age group—a brief communication. JACCOA.

[24] Kain ZN, Mayes LC, O'Connor TZ, Cicchetti DV (1996). Preoperative anxiety in children. Predictors and outcomes. Arch Pediatr Adolesc Med.

[25] Kim JE, Jo BY, Oh HM, Choi HS, Lee Y (2012). High anxiety, young age and long waits increase the need for preoperative sedatives in children. J Int Med Res.

[26] Perry JN, Hooper VD, Masiongale J (2012). Reduction of preoperative anxiety in pediatric surgery patients using age-appropriate teaching interventions. J Perianesth Nurs.

[27] Mali VP, Prabhakaran K, Yee KF, Hota S, Tang KP (2008). Elimination of attendance to children's emergency following circumcision in children.

[28] Pomicino L, Maccacari E, Buchini S (2018). Levels of anxiety in parents in the 24 hr before and after their child's surgery: a descriptive study. J Clin Nurs.

[29] Longard J, Twycross A, Williams AM, Hong P, Chorney J (2016). Parents' experiences of managing their child's postoperative pain at home: an exploratory qualitative study. J Clin Nurs.

[30] Andersson L, Johansson I, Österberg SA (2012). Parents' experiences of their child's first anaesthetic in day surgery. Br J Nurs.

[31] Lim SH, Mackey S, Liam JLW, He HG (2012). An exploration of Singaporean parental experiences in managing school-aged children's postoperative pain: a descriptive qualitative approach. J Clin Nurs.

[32] Sjöberg C, Svedberg P, Nygren JM, Carlsson IM (2017). Participation in paediatric perioperative care: 'what it means for parents'. J Clin Nurs.

[33] Ford K, Courtney-Pratt H, Fitzgerald M (2012). Post-discharge experiences of children and their families following children's surgery. J Child Health Care.

[34] Healy K (2013). A descriptive survey of the information needs of parents of children admitted for same day surgery. J Pediatr Nurs.

[35] Aranha PR, Dsouza SN (2019). Preoperative information needs of parents: a descriptive survey. J Res Nurs.

[36] Bogusaite L, Razlevice I, Lukosiene L, Macas A (2018). Evaluation of preoperative information needs in pediatric anesthesiology. Med Sci Monit.

[37] Nascimento LC, Warnock F, Pan R, Silva-Rodrigues FM, Castral TC, De Bortoli PS, de Moraes DC, Scochi CGS (2019). Parents' participation in managing their children's postoperative pain at home: an integrative literature review. Pain Manag Nurs.

[38] Gordon BK, Jaaniste T, Bartlett K, Perrin M, Jackson A, Sandstrom A, Charleston R, Sheehan S (2011). Child and parental surveys about pre-hospitalization information provision. Child Care Health Dev.

[39] Ruiz M, Rivers N, Pop RS (2012). Evaluating the effectiveness of the timing of postoperative education in the pediatric population. J Perianesth Nurs.

[40] Twycross A, Finley GA (2013). Children's and parents' perceptions of postoperative pain management: a mixed methods study. J Clin Nurs.

[41] Sng QW, Taylor B, Liam JL, Klainin-Yobas P, Wang W, He HG (2013). Postoperative pain management experiences among school-aged children: a qualitative study. J Clin Nurs.

[42] Sng QW, He HG, Wang W, Taylor B, Chow A, Klainin-Yobas P, Zhu L (2017). A meta-synthesis of children's experiences of postoperative pain management. Worldviews Evid Based Nurs.

[43] Chow CHT, Wan S, Pope E, Meng Z, Schmidt LA, Buckley N, Van Lieshout RJ (2018). Audiovisual interventions for parental preoperative anxiety: a systematic review and meta-analysis. Health Psychol.

[44] Buyuk ET, Bolişik B (2018). An analysis of the anxiety levels of mothers who participate in education and therapeutic games about their children's surgeries. J Perianesth Nurs.

[45] Chartrand J, Tourigny J, MacCormick J (2017). The effect of an educational pre-operative DVD on parents' and children's outcomes after a same-day surgery: a randomized controlled trial. J Adv Nurs.

[46] Berghmans J, Weber F, van Akoleyen C, Utens E, Adriaenssens P, Klein J, Himpe D (2012). Audiovisual aid viewing immediately before pediatric induction moderates the accompanying parents' anxiety. Paediatr Anaesth.

[47] Book F, Goedeke J, Poplawski A, Muensterer OJ (2020). Access to an online video enhances the consent process, increases knowledge, and decreases anxiety of caregivers with children scheduled for inguinal hernia repair: a randomized controlled study. J Pediatr Surg.

[48] Zhu L, Chan WCS, Liam JLW, Xiao C, Lim ECC, Luo N, Cheng KFK, He HG (2018). Effects of postoperative pain management educational interventions on the outcomes of parents and their children who underwent an inpatient elective surgery: a randomized controlled trial. J Adv Nurs.

[49] Fortier MA, Kain ZN (2015). Treating perioperative anxiety and pain in children: a tailored and innovative approach. Paediatr Anaesth.

[50] Wright KD, Raazi M, Walker KL (2017). Internet-delivered, preoperative, preparation program (I-PPP): development and examination of effectiveness. J Clin Anesth.

[51] Liu J, Zheng X, Zhang X, Feng Z, Song M, Lopez V (2020). The evidence and future potential of WeChat in providing support for Chinese parents of pediatric patients undergoing herniorrhaphy. J Transcult Nurs.

[52] Newton L, Sulman C (2018). Use of text messaging to improve patient experience and communication with pediatric tonsillectomy patients. Int J Pediatr Otorhinolaryngol.

[53] Yang JY, Lee H, Zhang Y, Lee JU, Park JH, Yun EK (2016). The effects of tonsillectomy education using smartphone text message for mothers and children undergoing tonsillectomy: a randomized controlled trial. Telemed J E Health.

[54] Ji L, Zhang X, Fan H, Han M, Yang H, Tang L, Shao Y, Lan Y, Li D (2016). drawMD APP-aided preoperative anesthesia education reduce parents anxiety and improve satisfaction. Patient Educ Couns.

[55] Bailey KM, Bird SJ, McGrath PJ, Chorney JE (2015). Preparing parents to be present for their child's anesthesia induction: a randomized controlled trial. Anesth Analg.

[56] Yu KE, Kim JS (2019). Effects of a posttonsillectomy management program using a mobile instant messenger on parents' knowledge and anxiety, and their children's compliance, bleeding, and pain. J Spec Pediatr Nurs.

[57] Kwan MK, Chiu CK, Gan CC, Chan CYW (2016). Can intraoperative text messages reduce parental anxiety of children undergoing posterior spinal fusion surgery for adolescent idiopathic scoliosis?. Spine (Phila Pa 1976).

[58] Cumino DO, Vieira JE, Lima LC, Stievano LP, Silva RAP, Mathias LAST (2017). Smartphone-based behavioural intervention alleviates children's anxiety during anaesthesia induction: a randomised controlled trial. Eur J Anaesthesiol.

[59] Seiden SC, McMullan S, Sequera-Ramos L, De Oliveira GS, Roth A, Rosenblatt A, Jesdale BM, Suresh S (2014). Tablet-based interactive distraction (TBID) vs oral midazolam to minimize perioperative anxiety in pediatric patients: a noninferiority randomized trial. Paediatr Anaesth.

[60] Stewart B, Cazzell MA, Pearcy T (2019). Single-blinded randomized controlled study on use of interactive distraction versus oral midazolam to reduce pediatric preoperative anxiety, emergence delirium, and postanesthesia length of stay. J Perianesth Nurs.

[61] Marechal C, Berthiller J, Tosetti S, Cogniat B, Desombres H, Bouvet L, Kassai B, Chassard D, de Queiroz Siqueira M (2017). Children and parental anxiolysis in paediatric ambulatory surgery: a randomized controlled study comparing 0.3 mg kg-1 midazolam to tablet computer based interactive distraction. Br J Anaesth.

[62] Liguori S, Stacchini M, Ciofi D, Olivini N, Bisogni S, Festini F (2016). Effectiveness of an app for reducing preoperative anxiety in children: a randomized clinical trial. JAMA Pediatr.

[63] Fernandes SC, Arriaga P, Esteves F (2014). Providing preoperative information for children undergoing surgery: a randomized study testing different types of educational material to reduce children's preoperative worries. Health Educ Res.

[64] Chow CHT, Van Lieshout RJ, Schmidt LA, Buckley N (2017). Tablet-based intervention for reducing children's preoperative anxiety: a pilot study. J Dev Behav Pediatr.

[65] Bandura A (1997). Self-Efficacy: The Exercise of Control.

[66] Peterson AM, Harper FWK, Albrecht TL, Taub JW, Orom H, Phipps S, Penner LA (2014). Parent caregiver self-efficacy and child reactions to pediatric cancer treatment procedures. J Pediatr Oncol Nurs.

[67] Jones TL, Prinz RJ (2005). Potential roles of parental self-efficacy in parent and child adjustment: a review. Clin Psychol Rev.

[68] Charana A, Tripsianis G, Matziou V, Vaos G, Iatrou C, Chloropoulou P (2018). Preoperative anxiety in Greek children and their parents when presenting for routine surgery. Anesthesiol Res Pract.

[69] Chieng YJS, Chan WCS, Klainin-Yobas P, He HG (2014). Perioperative anxiety and postoperative pain in children and adolescents undergoing elective surgical procedures: a quantitative systematic review. J Adv Nurs.

[70] Chahal N, Manlhiot C, Colapinto K, Van Alphen J, McCrindle BW, Rush J (2009). Association between parental anxiety and compliance with preoperative requirements for pediatric outpatient surgery. J Pediatr Health Care.

[71] Kampouroglou G, Velonaki V, Pavlopoulou I, Drakou E, Kosmopoulos M, Kouvas N, Tsagkaris S, Fildissis G, Nikas K, Tsoumakas K (2020). Parental anxiety in pediatric surgery consultations: the role of health literacy and need for information. J Pediatr Surg.

[72] Chorney JM, Twycross A, Mifflin K, Archibald K (2014). Can we improve parents' management of their children's postoperative pain at home?. Pain Res Manag.

[73] Hoffmann TC, Glasziou PP, Boutron I, Milne R, Perera R, Moher D, Altman DG, Barbour V, Macdonald H, Johnston M, Lamb SE, Dixon-Woods M, McCulloch P, Wyatt JC, Chan AW, Michie S (2014). Better reporting of interventions: Template for Intervention Description and Replication (TIDieR) checklist and guide. BMJ.

[74] Elo S, Kyngäs H (2008). The qualitative content analysis process. J Adv Nurs.

[75] Hsieh HF, Shannon SE (2005). Three approaches to qualitative content analysis. Qual Health Res.

[76] Lincoln YS, Guba EG (1985). Naturalistic Inquiry.

[77] Hashemi SB, Amirfakhraei A, Mosallanezhad M, Amiri A (2019). The effect of education on anxiety and self-efficacy in mothers of 1-3-year-old children under cochlear implant surgery, 2018: a randomised controlled clinical trial. Rev Latinoam de Hipertens.

[78] Cheng F (2018). Build Mobile Apps with Ionic 4 and Firebase: Hybrid Mobile App Development. 2nd Edition.

[79] (2015). Children's use of mobile phones: an international comparison 2015. Global System for Mobile Communications Association.

[80] Galehantomo GPS (2015). Platform comparison between games console, mobile games and PC games. Eur J Inf Syst.

[81] Law GC, Neihart M, Dutt A (2018). The use of behavior modeling training in a mobile app parent training program to improve functional communication of young children with autism spectrum disorder. Autism.

[82] Quelly SB, Norris AE, DiPietro JL (2016). Impact of mobile apps to combat obesity in children and adolescents: a systematic literature review. J Spec Pediatr Nurs.

[83] Ventola CL (2014). Mobile devices and apps for health care professionals: uses and benefits. P T.

[84] Hoddinott P (2015). A new era for intervention development studies. Pilot Feasibility Stud.

