# Supplementary material for: An Intelligent Customer-Driven Digital Solution to Improve Perioperative Health Outcomes Among Children Undergoing Circumcision and Their Parents: Development and Evaluation
Source: JMIR Form Res. 2024 Feb 16;8:e52337. doi: 10.2196/52337 (PMC10907943; doi:10.2196/52337)
Supplement: Multimedia Appendix 1 [file formative_v8i1e52337_app1.docx]

Appendix 1: Interview guide

Please fill in the respective blanks with your honest opinion.

BuddyCare

1. What did you like about the app?
2. What did you not like about the app?
3. Was the app easy to use/navigate? How so or how not?
4. Do you think the app is useful? How so or how not?
5. Any suggestions for improvements?

Triumf Health

1. What did you like about the app?
2. What did you not like about the app?
3. Was the app easy to use/navigate? How so or how not?
4. Do you think the app is useful? How so or how not?
5. Any suggestions for improvements?
