# Supplementary material for: An Intelligent Customer-Driven Digital Solution to Improve Perioperative Health Outcomes Among Children Undergoing Circumcision and Their Parents: Development and Evaluation
Source: JMIR Form Res. 2024 Feb 16;8:e52337. doi: 10.2196/52337 (PMC10907943; doi:10.2196/52337)
Supplement: Multimedia Appendix 2 [file formative_v8i1e52337_app2.docx]

|  | Timepoint | Information |
| --- | --- | --- |
| Pre-op | Preoperative  Day 1 | Welcome message |
|  | Preoperative  Day 2 | **Overview of Circumcision in children**   - Risks of male circumcision - Tips on how to explain the procedure to your child |
|  | Preoperative  Day 3 | **Post-operative pain**   - What is postoperative pain? - So how do you assess your child’s pain? How to know whether it is severe or mild or no pain?   - Faces Pain Scale-Revised |
|  | Preoperative  Day 4 | **How to help your child to cope with his pain?**  1) Medications  Painkillers/Medicines to relieve pain and make my child comfortable |
|  | Preoperative  Day 5 | **How to help your child to cope with his pain?**  I am worried about too much medication. Can I make my child comfortable without having to give painkiller medicines? |
|  | Preoperative  Day 6 | **Positive Reinforcement**   - What is it? - How does it work? - How to do it? |
|  | Preoperative  Day 7 | **Breathing Techniques**  How to do it? |
|  | Preoperative  Day 8 | **Distraction**   - What is it? - How to do it? |
|  | Preoperative  Day 9 | **Emotional Support by parents and/or caregivers**   - Presence - Touch - Comforting and reassurance   **Summary** |
|  | Day 10 | **Practice (breathing techniques)** |
|  | Day 11 | **What to expect about anaesthesia?** |
|  | Day 12 | **Practice (breathing techniques and positive reinforcement)** |
|  | Day 13 | **Preparing for the Circumcision** |
|  | Day 14 | **Pre-operative Instructions**   - What to bring to the hospital - Pre-operation instructions - Positive Quote |
|  | Operation day | **Before the procedure**  **Recovering after male circumcision**   - When to contact the hospital   **Positive Quote** |
| Post-op | 1st day after circumcision | **Wound Management**   - What you would need to clean the wound - How to clean - Positive Quote   **When to bring your child to the emergency** |
|  | POD 2 | **Managing your son’s pain** |
|  | POD 3 | **Managing your son’s pain** |
|  | POD 4 |  |
|  | POD 5 |  |
|  | POD 6 |  |
|  | POD 7 |  |
|  | POD 8 |  |
|  | POD 9 |  |
|  | POD 10 |  |
|  | POD 11 |  |
|  | POD 12 |  |
|  | POD 13 |  |
|  | POD 14 | **Thank you for your message** |
