# Supplementary material for: An Intelligent Customer-Driven Digital Solution to Improve Perioperative Health Outcomes Among Children Undergoing Circumcision and Their Parents: Development and Evaluation
Source: JMIR Form Res. 2024 Feb 16;8:e52337. doi: 10.2196/52337 (PMC10907943; doi:10.2196/52337)

Pre-operative Instructions

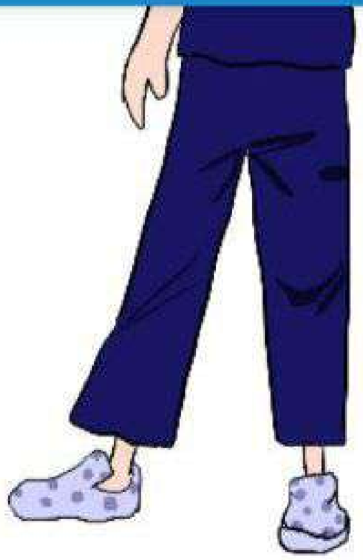

Here's a quote that we hope it will be of help to you!

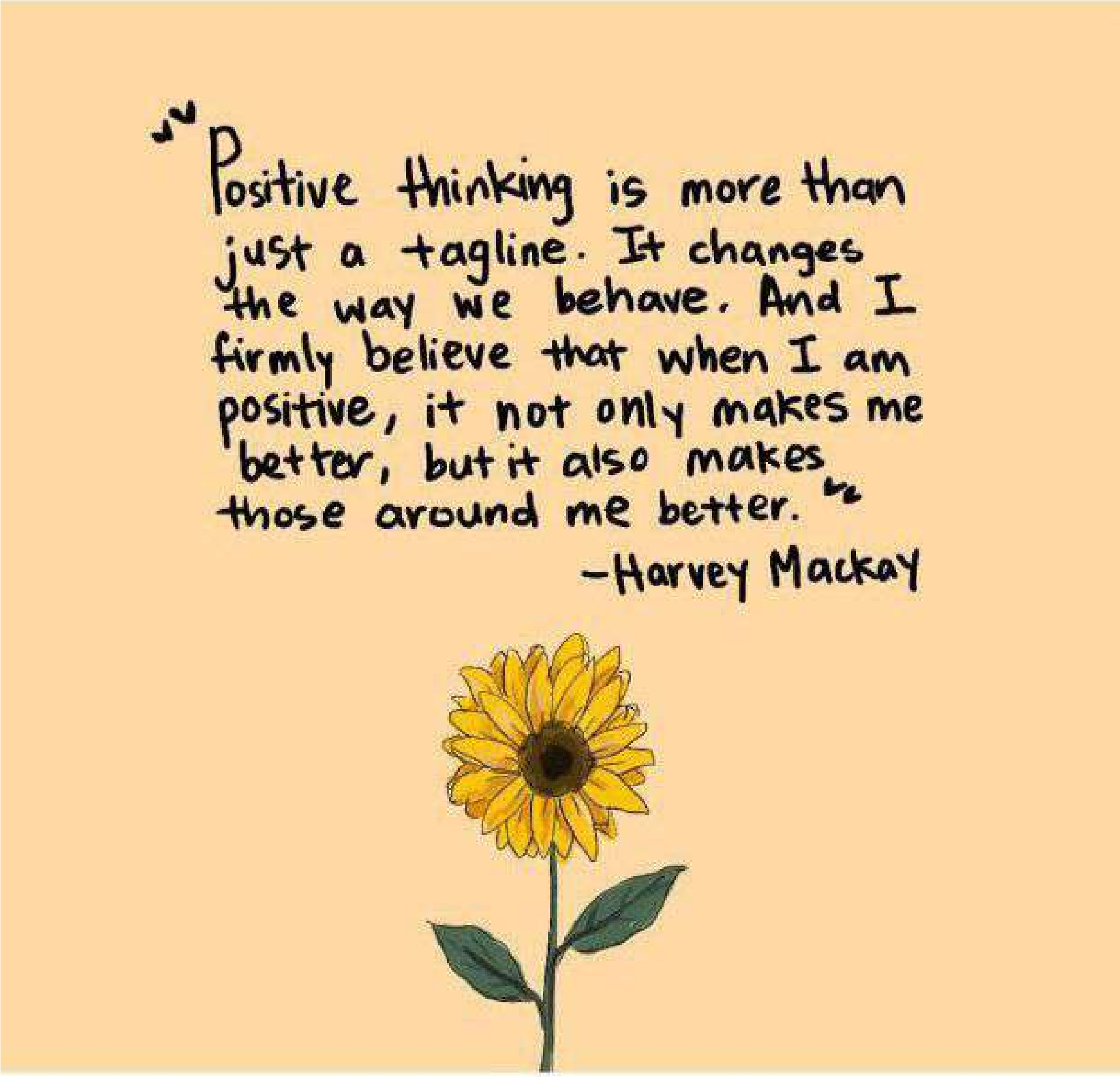

Supplement: Multimedia Appendix 3 [file formative_v8i1e52337_app3.pdf]
