# Supplementary figures and images for: An Intelligent Customer-Driven Digital Solution to Improve Perioperative Health Outcomes Among Children Undergoing Circumcision and Their Parents: Development and Evaluation
Source: JMIR Form Res. 2024 Feb 16;8:e52337. doi: 10.2196/52337 (PMC10907943; doi:10.2196/52337)

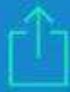

# Menu

12/25/19, Pediatric Circumcision

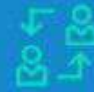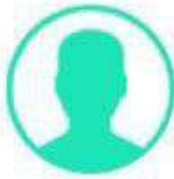

zy k

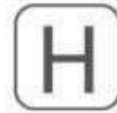

Logo

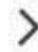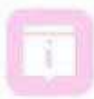

Info Package

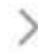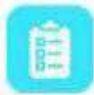

Forms

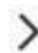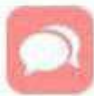

Messages

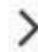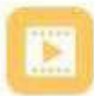

Videos

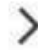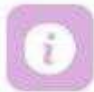

About

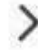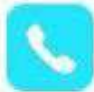

Contact

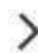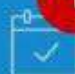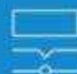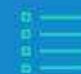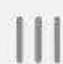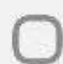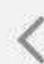

Supplement: Multimedia Appendix 4 [file formative_v8i1e52337_app4.pdf]
