# Supplementary material for: An Intelligent Customer-Driven Digital Solution to Improve Perioperative Health Outcomes Among Children Undergoing Circumcision and Their Parents: Development and Evaluation
Source: JMIR Form Res. 2024 Feb 16;8:e52337. doi: 10.2196/52337 (PMC10907943; doi:10.2196/52337)
Supplement: Multimedia Appendix 5 [file formative_v8i1e52337_app5.pdf]

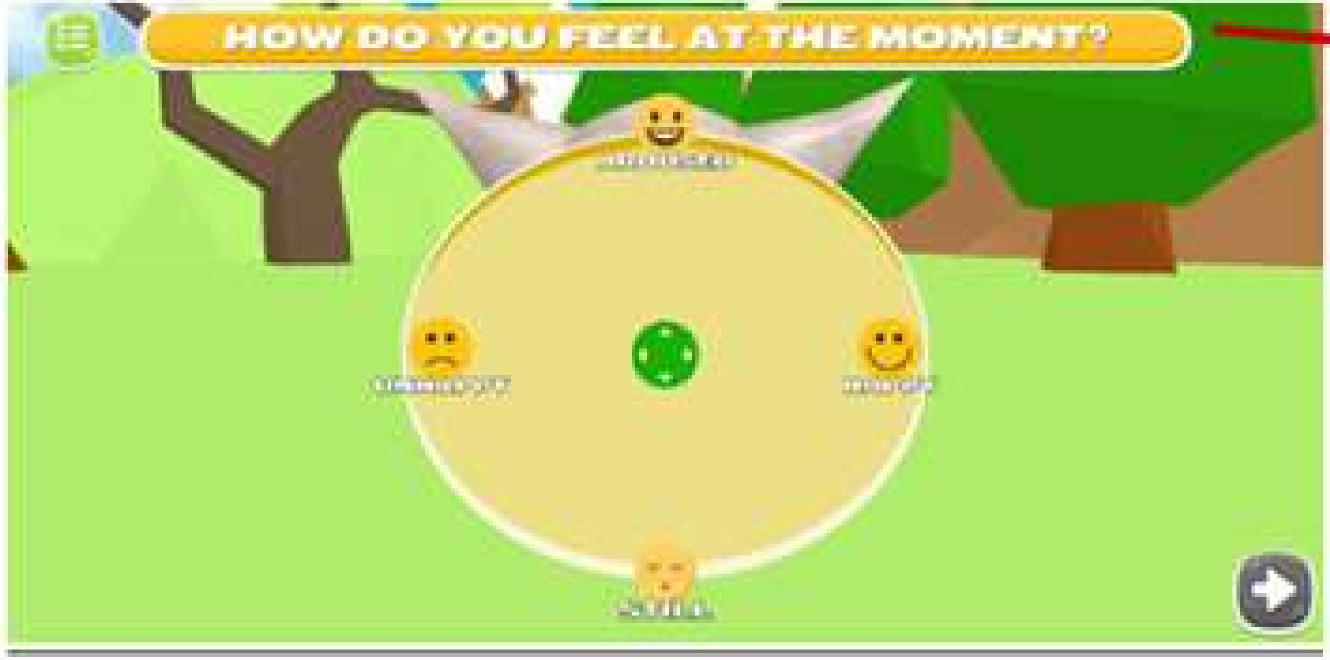

The child was able to indicate how he was feeling using this circumplex model and the game gave appropriate psychological support to the child according to the child's response. E.g. if he indicated that he was unhappy, the game gives small tips to cheer the child up.
